# Tissue-associated and vertically transmitted bacterial symbiont in the coral *Pocillopora acuta*

**DOI:** 10.1093/ismejo/wrad027

**Published:** 2024-01-10

**Authors:** Justin Maire, Sarah Jane Tsang Min Ching, Katarina Damjanovic, Hannah E Epstein, Louise M Judd, Linda L Blackall, Madeleine J H van Oppen

**Affiliations:** School of BioSciences, The University of Melbourne, Parkville, 3010 VIC, Australia; School of BioSciences, The University of Melbourne, Parkville, 3010 VIC, Australia; School of BioSciences, The University of Melbourne, Parkville, 3010 VIC, Australia; Australian Institute of Marine Science, PMB No 3, Townsville, 4810 QLD, Australia; Australian Institute of Marine Science, PMB No 3, Townsville, 4810 QLD, Australia; ARC Centre of Excellence for Coral Reef Studies, James Cook University, Townsville, 4811 QLD, Australia; School of Life Sciences, University of Essex, Wivenhoe Park, Colchester, Essex CO4 3SQ, United Kingdom; Doherty Applied Microbial Genomics, Department of Microbiology and Immunology, The University of Melbourne at the Peter Doherty Institute for Infection and Immunity, Parkville, 3010 VIC, Australia; School of BioSciences, The University of Melbourne, Parkville, 3010 VIC, Australia; School of BioSciences, The University of Melbourne, Parkville, 3010 VIC, Australia; Australian Institute of Marine Science, PMB No 3, Townsville, 4810 QLD, Australia

**Keywords:** Endozoicomonas, Endozoicomonadaceae, Cnidaria, symbiosis, microbiome, genomics

## Abstract

Coral microhabitats are colonized by a myriad of microorganisms, including diverse bacteria which are essential for host functioning and survival. However, the location, transmission, and functions of individual bacterial species living inside the coral tissues remain poorly studied. Here, we show that a previously undescribed bacterial symbiont of the coral *Pocillopora acuta* forms cell-associated microbial aggregates (CAMAs) within the mesenterial filaments. CAMAs were found in both adults and larval offspring, suggesting vertical transmission. In situ laser capture microdissection of CAMAs followed by 16S rRNA gene amplicon sequencing and shotgun metagenomics produced a near complete metagenome-assembled genome. We subsequently cultured the CAMA bacteria from *Pocillopora acuta* colonies, and sequenced and assembled their genomes. Phylogenetic analyses showed that the CAMA bacteria belong to an undescribed *Endozoicomonadaceae* genus and species, which we propose to name *Candidatus* Sororendozoicomonas aggregata gen. nov sp. nov. Metabolic pathway reconstruction from its genome sequence suggests this species can synthesize most amino acids, several B vitamins, and antioxidants, and participate in carbon cycling and prey digestion, which may be beneficial to its coral hosts. This study provides detailed insights into a new member of the widespread *Endozoicomonadaceae* family, thereby improving our understanding of coral holobiont functioning. Vertically transmitted, tissue-associated bacteria, such as *Sororendozoicomonas aggregata* may be key candidates for the development of microbiome manipulation approaches with long-term positive effects on the coral host.

Corals associate with diverse bacteria with essential functions, such as protection against pathogens and nutrient cycling [1]. Among these bacteria, a small portion resides within the coral tissues [2], sometimes forming cell-associated microbial aggregates (CAMAs) [3-6]. However, the identification and characterization of CAMA-forming bacteria in corals remain in its infancy. Bacteria of the *Endozoicomonas* genus are well known to form CAMAs [3, 4, 7], and are widespread coral symbionts generally considered to be mutualistic through sulfur and phosphorus cycling, and amino acid and B vitamin synthesis [3, 7-10]. *Simkania* sp. (*Chlamydiota*) was recently shown to also form CAMAs in *Pocillopora acuta* from Feather Reef (Great Barrier Reef [GBR], Australia; Fig. S1) [3], and *Chlamydia*- and *Rickettsia*-like inclusions were detected in *Acropora muricata* [5], although their functions remain unclear.

Here, we characterized CAMAs present in *P. acuta* colonies from Orpheus Island in the central GBR. *Pocillopora acuta* is an asexual brooder, producing fully formed, genetically identical larvae, and a sexual broadcast spawner, releasing gametes into the water column for external fertilization [11]. In a previous study, the microbiome of three Orpheus Island colonies was assessed at three different life stages, finding that *Endozoicomonas* is highly abundant in larvae (5%–35%), recruits (10%–40%), and adults (40%–80%) [6]. Detection of CAMAs in adults and larvae indicated possible vertical transmission [6]. In a separate study, monitoring of 12 colonies from the same site throughout an entire year showed high abundances of *Endozoicomonas* (5%–70%), highlighting the stability of the association despite environmental fluctuations [12]. Using samples from these two studies (Fig. S2A and B), we verified by fluorescence in situ hybridization (FISH) that both adults (X7 colony, three branches from the one colony [12]) and asexually produced larvae (OI2 and OI3 colonies, three larvae from each of the two colonies [6]) possessed CAMAs (Fig. 1A–E). In X7 adult polyps, CAMAs localized to the mesenterial filaments (Fig. 1A–C). In OI2 and OI3 larvae, CAMAs were found in the gastrodermis (Fig. 1D and E).

**Figure 1:**
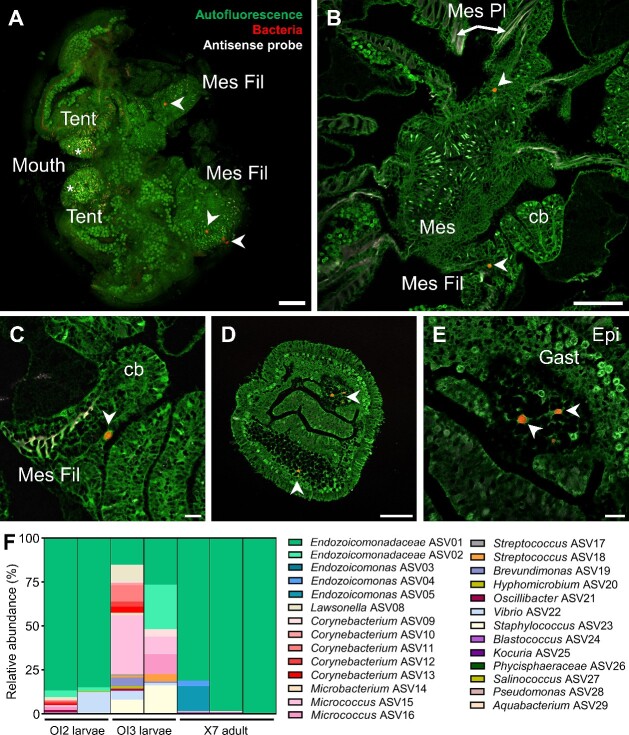
Location and identity of CAMAs in Orpheus Island *P. acuta*. (A) CAMAs in an adult polyp (X7 colony) by whole-mount FISH. (B–E) FISH images showing the location of CAMAs in sectioned adult polyps (B, C; X7 colony) and sectioned larvae (D, E; OI2 colony). White arrowheads point at CAMAs. Green: autofluorescence; red: EUB338-mix probe (all bacteria); white: non-EUB probe (negative control). Note the nonspecific binding in tentacles (red and white signal overlapping, white asterisks), often localized in nematocysts. Tent: tentacle; Mes Fil: mesenterial filaments; Mes: mesenteries; Mes Pl: mesogleal plates; cb: cnidoglandular band; Gast: gastrodermis; Epi: epidermis. Scale bars: 100 μm for A, B, D; 20 μm for C and E. **(**F) Relative abundance of bacterial ASVs in CAMAs of larvae (OI2 and OI3 colonies) and adult branches (X7 colony) isolated by LCM. Each bar represents one replicate (either three pooled larvae or one adult branch). Detailed composition is available in Table S2.

To taxonomically identify the bacteria present in the CAMAs, we excised CAMAs using laser capture microdissection (LCM), followed by 16S rRNA gene amplicon sequencing, in three replicate branches of the X7 colony and two replicates of three pooled larvae from each of the OI2 and OI3 colonies (Fig. S3, Table S1). Two ASVs assigned to *Kistimonas* (*Endozoicomonadaceae* ASVs 01 and 02) made up most of the reads in OI2 larvae and X7 branches (Fig. 1F, Table S2). Even though the samples were collected at the same site, they originated from different host colonies and were collected several months apart, highlighting the temporal stability of this association among individuals and through ontogeny. *Kistimonas* is part of the *Endozoicomonadaceae* family along with *Endozoicomonas*. Because of taxonomic discrepancies discussed below, we refer to these two ASVs as *Endozoicomonadaceae* ASVs 01 and 02. The two ASVs were also present in OI3 larvae at a lower relative abundance. In addition, three *Endozoicomonas* ASVs were found in X7 and were identical to *Endozoicomonas* present in *P. acuta* from Feather Reef [3]. FISH using an *Endozoicomonadaceae* probe provided additional support for this taxonomy in both adult and larval CAMAs (Fig. S4).

Although *Kistimonas* was not reported in the original studies [6, 12], a reanalysis of both previous studies reassigned three *Endozoicomonas* ASVs to *Kistimonas* (Fig. S5, Table S3), two of which were 100% identical to *Endozoicomonadaceae* ASVs 01 and 02 found in our excised CAMA samples. Virtually no *Endozoicomonas* strains (only one ASV at 0.02%–0.04% relative abundance) were detected in whole larvae or recruits in our reanalysis of the earlier datasets (Fig. S5), suggesting that *Endozoicomonas* are acquired horizontally in Orpheus Island *P. acuta*. Conversely, *Endozoicomonadaceae* ASVs 01 and 02 were present at all life stages (i.e. larvae, recruits, and adults of the same colonies, Fig. S5) and formed CAMAs, supporting their vertical transmission during asexual reproduction. Whether whole CAMAs or individual bacteria are transmitted from parent colony to asexually produced larvae remains unknown. The possibility of vertical transmission in sexually produced offspring (i.e. through the production of gametes) remains to be investigated. Strong evidence for vertical transmission of bacteria in corals has only been reported twice before [13, 14].

Shotgun sequencing of the pooled X7 CAMA samples yielded one metagenome-assembled genome (MAG) (Pac_X7, 95.9% complete, Table S4), the 16S rRNA gene sequence of which was identical to *Endozoicomonadaceae* ASV01. An additional *P. acuta* colony (P3, Fig. S2C) was collected from Orpheus Island in June 2023, from which we cultured 10 bacterial isolates with 16S rRNA gene sequences identical to Pac_X7, one of which was whole-genome sequenced (Pac_P3-11-1, 97.1% complete, Table S4). Both assemblies had 99.8% Average Nucleotide Identity (ANI) and 99.6% Average Amino acid Identity (AAI) (Fig. S6), confirming that the Pac_P3-11-1 isolate is indeed a CAMA bacterium. Phylogenetic analysis of 120 marker genes placed both genomes separately from all described *Endozoicomonadaceae* genera (Fig. 2A, Table S5), along with *Endozoicomonadaceae* SCSIO12664 (99.6% 16S rRNA gene sequence identity, 99.2% ANI, 98.8% AAI), isolated from *Pocillopora damicornis* in the South China Sea [15]. This was supported by 16S rRNA gene phylogeny (Fig. S7). Together with low AAI (highest score of 60.1% with *Endozoicomonas elysicola*, Fig. S6) and low 16S rRNA gene identity (highest score of 93.97% with *Endozoicomonas gorgoniicola*), these data support the placement of Pac_P3-11-1 and *Endozoicomonadaceae* SCSIO12664 in a new genus and species, which we propose to name *Candidatus* Sororendozoicomonas aggregata, gen. nov., sp. nov (here referred to as *Sororendozoicomonas aggregata*; see description in the Supplementary Material).

**Figure 2:**
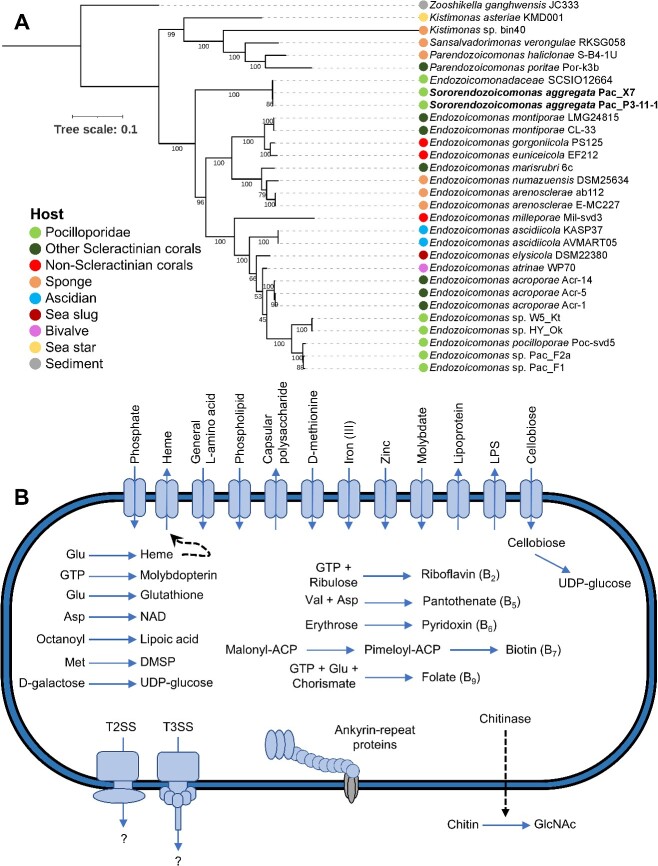
Taxonomy and functional potential of *S. aggregata*, CAMA member of Orpheus Island *P. acuta*. (A) Maximum likelihood phylogeny of *Endozoicomonadaceae* based on 120 marker genes and 28 *Endozoicomonadaceae* genomes in addition to the two genomes recovered in this study. *Zooshikella* was chosen as the outgroup. Bootstrap support values based on 1000 replications are provided. Additional data on the reference genomes are available in Table S5. (B) Overview of the metabolic potential from the genome sequence of Pac_P3-11-1 recovered from *P. acuta* CAMAs. The pathways represented here are at least 80% complete (Fig. S7). Dashed arrows represent hypotheticals. T2SS: Type II secretion system; T3SS: Type III secretion system; DMSP: dimethylsulfoniopropionate; LPS: lipopolysaccharide; NAD: nicotinamide adenine dinucleotide; GlcNAc: N-acetylglucosamine.

Genome annotation (Figs 2B and S8, Table S6) revealed Pac_P3-11-1 encodes several genes indicative of host–symbiont interactions, including Types II and III secretion systems, 35 eukaryotic-like proteins (Table S7), and three secondary metabolites with putative antimicrobial activity (Table S8). This gene repertoire is similar to that of other coral-associated *Endozoicomonadaceae* [3, 4, 8-10] and may regulate host colonization, aggregation, and vertical transmission. Additionally, we recovered pathways for the biosynthesis of all amino acids (except phenylalanine, tyrosine, and arginine), as well as riboflavin (vitamin B_2_), pantothenate (B_5_), pyridoxine (B_6_), biotin (B_7_), and folate (B_9_), which could assist with coral metabolism [3, 4, 9, 10, 16]. Pac_P3-11-1 also shows potential for the scavenging of reactive oxygen species, which are believed to drive coral bleaching when present in excess [17], as it possesses pathways for the synthesis of the antioxidants heme, lipoic acid, and glutathione, and the *dsyB* gene, essential for the synthesis of dimethylsulfoniopropionate [18]. Finally, we uncovered high potential for carbon cycling, with a full pathway for the degradation of D-galactose into UDP-glucose, a cellobiose phosphotransferase system (*celABC*) and glucosidase (*celF*) that can import extracellular cellobiose and degrade it into UDP-glucose, and an endo-chitinase that can degrade chitin into N-acetylglucosamine. Cellobiose is a major cell wall component of some algae, including *Symbiodiniaceae*, and chitin is a major cell wall component of zooplankton and crustaceans, all potential food sources for corals. Endo-chitinases were recently found to be widespread in *Endozoicomonadaceae* and are potentially exported [19]. Both sets of enzymes may therefore assist the coral host with prey digestion and provide carbon- and nitrogen-rich oligo-sugars to the holobiont.

In conclusion, we described and cultured the first coral tissue-associated, vertically transmitted member of the *Endozoicomonadaceae* family. *Sororendozoicomonas* was not found in other GBR *P. acuta* populations that harbor *Endozoicomonas* [3], suggesting geographic location may impact *Endozoicomonadaceae* associations. Additionally, *Endozoicomonas* CAMAs were previously found in pocilloporid tentacles [3, 4], contrasting with *Sororendozoicomonas*’ location in the mesenterial filaments, where it could aid in digestion and nutrient acquisition from host heterotrophic feeding. Other potentially beneficial functions, such as vitamin synthesis or antioxidant abilities, may also increase host fitness. This study provides additional insights into the wide array of functions, benefits, and lifestyles of *Endozoicomonadaceae* within coral holobionts and reinforces the ecological importance of this bacterial family.

## Supplementary Material

Supplementary_information_revision_wrad027

Table_S2-raw_values_CAMAs_wrad027

Table_S3-raw_values_Endoz_in_Kat_Hannah_wrad027

Table_S5-reference_genomes_wrad027

Table_S6-bakta_wrad027

## Data Availability

Raw data are available under NCBI BioProject IDs PRJNA891898 (larvae CAMAs, MiSeq raw data) and PRJNA974967 (adult CAMAs, MiSeq and NovaSeq raw data, Pac_X7 MAG assembly; Pac_P3-11-1 NextSeq raw reads and assembly). The complete 16S rRNA gene sequence of Pac_P3-11-1 is available under the accession number OR505856.
