# Supplementary material for: Tissue-associated and vertically transmitted bacterial symbiont in the coral Pocillopora acuta
Source: ISME J. 2024 Jan 10;18(1):wrad027. doi: 10.1093/ismejo/wrad027 (PMC10833068; doi:10.1093/ismejo/wrad027)
Supplement: Supplementary_information_revision_wrad027 [file supplementary_information_revision_wrad027.docx]

**Description of *Sororendozoicomonas* gen. nov.**

*Sororendozoicomonas* (So.ror.en.do.zo.i.co.mo’nas. L. fem. n. soror, sister; *Endozoicomonas*, taxonomic name of a bacterial genus; *Sororendozoicomonas* referring to the close relationship to the bacterial genus *Endozoicomonas*). The name was registered through SeqCode [1].

The genus *Sororendozoicomonas* represents a distinct lineage within the *Endozoicomonadaceae* family, sister to *Endozoicomonas*, supported by concatenated marker gene phylogenies (Figures 2A) as well as 16S rRNA gene phylogeny (Figure S7) in the present study. *Sororendozoicomonas* show similar functional and metabolic potential to *Endozoiocomonas* species (Figure 2B). The new genus contains two strains isolated from *Pocillopora* corals (Pac-P3-11-1, from the present study, and SCSIO12664 [2]) and potentially additional strains isolated from Red Sea *Stylophora pistillata* [3] (only 16S rRNA gene sequences are available - KC669256.1, KC669230.1, KC669217.1, KC669131.1).

**Description of *Sororendozoicomonas aggregata* sp. nov.**

*Sororendozoicomonas aggregata* (ag.gre.ga'ta. L. fem. part. adj. *aggregata,* joined together, referring to the ability to form aggregates within coral tissues). The name was registered through SeqCode [1]. The new species contains two strains isolated from *Pocillopora* corals (Pac-P3-11-1, from the present study, and SCSIO12664 [2]).

Type strain: Pac_P3-11-1, isolated from tissues of the coral *Pocillopora acuta*, collected from Orpheus Island, Australia (18°36'16.26" S, 146°29'24.27" E). Taxonomic assignment based on concatenated marker gene phylogenies (Figure 2A) as well as 16S rRNA gene phylogeny (Figure S7). Grows on Marine Agar 2216 (Difco) between 23°C and 28°C, forming circular, white, raised, sticky colonies with entire margins.

**Material and methods**

**Coral larvae collection (OI2 and OI3 colonies)**

For OI2 and OI3, parent colonies were collected in 2017 from Orpheus Island (Little Pioneer Bay; 18°36'3.6"S 146°29'20.4"E), in the central Great Barrier Reef in Australia (Figure S1 and S2A), and maintained and sampled as part of a previous experiment [4]. Briefly, before planulation, colonies were maintained in individual acrylic aquaria that received indirect natural sunlight and 0.4 μm-filtered seawater. A filter was fitted at each outlet of the acrylic tank to collect released planulae. Larvae were washed with 0.22-µm filtered seawater (FSW), transferred from the filter into a microcentrifuge tube using a sterile pipette tip, and as much water as possible was removed from the tube without disturbing the larvae. Larvae were fixed for 10 hrs at 4°C in 4% paraformaldehyde (PFA) prepared in FSW, rinsed twice in FSW, and stored in 50% ethanol-PBS at -20°C. Around 10 larvae per parent colony were fixed.

**Adult coral collection (X7 and P3 colonies)**

For the X7 colony (Figure S2B), coral fragments were sampled from the field in 2016 from the same site at Orpheus Island as part of a previous experiment (Figure S1) [5]. From this study, only samples collected in November 2016 were processed here. Four small coral fragments (2-5 cm in length) were snapped off the colonies with forceps, fixed for 24 hrs in 4% PFA in FSW, rinsed twice in FSW, and stored in 50% ethanol-PBS at -20°C. Following fixation, coral branches were decalcified in EDTA 10%. EDTA was renewed every two days, and samples were kept at 4°C on a rotating wheel, until there was no skeleton left (around two weeks). Samples were then rinsed in PBS 1X, and stored at 4°C in PBS 1X.

For the P3 colony (Figure S2C), coral colonies were collected at 1-4 m depth with a chisel and hammer from Orpheus Island (Little Pioneer Bay, 18°36'16.26" S, 146°29'24.27" E, Collection permit G22/46479.1) in June 2023. Four colonies were brought back to the Australian Institute of Marine Science for overnight holding before being shipped to the University of Melbourne the next day. On arrival, the corals were placed in 130 L recirculating system tanks and kept there overnight for 10 h at 25°C in 35 p.p.t. reconstituted sea water (Red Sea Salt, R11065, Red Sea, USA).

**Fluorescence *in situ* Hybridization (sections)**

Sample processing, embedding, and sectioning (3-µm thickness) were performed by the Melbourne Histology Platform (University of Melbourne) as previously described [6]. Fluorescence *in situ* hybridization (FISH) was then performed as previously described [4], but final probe concentration during hybridization was 5 ng/µL. See Table S9 for probe sequences, fluorophores, and formamide concentrations. Slides were mounted in CitiFluor CFM3 mounting medium (proSciTech, Australia), covered with a coverslip and sealed with clear nail polish. Slides were kept at 4°C until observation.

**Fluorescence *in situ* Hybridization (whole-mount)**

Single polyps of the X7 colony were dissected in PBS 1X using Dumont tweezers under a dissecting microscope. Samples were cleared of autofluorescence by incubating polyps in methanol-PBS 1X (50:50 v/v) for 10 min, methanol-PBS 1X (75:25 v/v) for 10 min, methanol-PBS 1X (90:10 v/v) for 10 min, 100% methanol for 10 min, methanol-PBS 1X + Triton X-100 0.2% (90:10 v/v) for 10 min, methanol-PBS 1X + Triton X-100 0.2% (75:25 v/v) for 10 min, methanol-PBS 1X + Triton X-100 0.2% (50:50 v/v) for 10 min, PBS 1X + 0.2% Triton X-100 for 10 min, and stored at 4°C in PBS 1X. FISH was performed on whole polyps as previously described [6], with the probes shown in Table S9**.** Before observation, single polyps were cut in half to expose the gastric cavity and mesenteries, and deposited onto 8-well coverslip-bottom slides (ibidi, USA) with a drop of milliQ water to avoid drying.

**Confocal Laser Scanning Microscopy**

Slides were observed on a Nikon A1R confocal laser scanning microscope (Nikon, Japan) with the NIS325 Element software. Virtual band mode was used to acquire variable emission bandwidth to tailor acquisition for specific fluorophores. The fluorophores Atto550 were excited using the 561 nm laser line, Atto647 using the 640 nm laser line, and the coral autofluorescence using the 488 nm laser line with a detection range of 570-620 nm for Atto550, 660-710 nm for Atto647, and 500-550 nm for coral autofluorescence. For three-dimensional reconstructions of Z-stacks (for whole polyps), sections were acquired using Z steps of 3.2 μM with the 10X objective. Nd2 files were processed using ImageJ. Z-stacks were projected in two-dimensional images using the ‘Max Intensity’ projection type. Linear adjustments of brightness and contrast were performed when necessary and applied to the entire image and to each channel independently. Channels were then given artificial colors (see figure legends) and merged.

**Laser Capture Microdissection of CAMAs and DNA extraction**

Laser capture microdissection (LCM) of CAMAs in OI2, OI3, and X7 samples was performed as previously described [6]. For OI2 and OI3 larvae, three replicates each containing three larvae were processed. For each replicate, ten slides each containing eight sections were processed (~80 3-µm sections per replicate in total). For X7 branches, three replicates each containing one branch were processed. For each replicate, 15 slides each containing four sections were processed (~60 3-µm sections per replicate in total). Tissue areas without CAMAs were also separately captured as a negative control. DNA extraction was then conducted as previously described [6], using the Arcturus® PicoPure® DNA Extraction Kit (Applied Biosystems, USA). Three caps containing no captured tissue, but that were open in the LCM facility to capture air contamination, were also included as extraction blanks.

**16S rRNA gene amplicon sequencing**

Hypervariable regions V5-V6 of the 16S rRNA genes were amplified using the primer set 784F (5ʹ GTGACCTATGAACTCAGGAGTCAGGATTAGATACCCTGGTA 3ʹ) and 1061R (5ʹ CTGAGACTTGCACATCGCAGCCRRCACGAGCTGACGAC 3ʹ). Adapters were attached to the primers and are underlined. Bacterial 16S rRNA genes were PCR-amplified on a SimpliAmp Thermal Cycler (Applied Biosystems, ThermoFisher Scientific). Each reaction contained 1 μL of DNA template, 1.5 μL of forward primer (10 μM stock), 1.5 μL of reverse primer (10 μM stock), 7.5 μL of 2x QIAGEN Multiplex PCR Master Mix (Qiagen, Germany) and 3.5 μL of nuclease-free water (Thermofisher), with a total volume of 15 μL per reaction. Three triplicate PCRs were conducted for each sample and three no-template PCRs were conducted as negative controls. PCR conditions for the 16S rRNA genes were as follows: initial denaturation at 95°C for 3 min, then 18 cycles of: denaturation at 95°C for 15 s, annealing at 55°C for 30 s, and extension at 72°C for 30 s; with a final extension at 72°C for 7 minutes. Samples were then held at 4°C. Following PCR, triplicates were pooled, resulting in 45 μL per sample. Amplicon sequencing library preparation was conducted as previously described [7] and sequencing was performed at the Walter and Eliza Hall Institute (WEHI) in Melbourne, Australia on one MiSeq V3 system (Illumina) with 2x300bp paired-end reads.

**16S rRNA gene amplicon sequencing analysis**

QIIME2 v 2021.8 [8] was used for processing 16S rRNA gene sequences. The plugin demux [8] was used to create an interactive plot to visualize the data and assess the quality, for demultiplexing and quality filtering of raw sequences. The plugin cutadapt [9] was used to remove the primers and MiSeq adapters. DADA2 [10] was used for denoising and chimera checking, trimming, dereplication, generation of a feature table, joining of paired-end reads, correcting sequencing errors, and removing low quality reads (Q-score < 30). Summary statistics were obtained using the feature-table to ensure processing was successful. Taxonomy was assigned by training a naive Bayes classifier with the feature-classifier plugin [8], based on a 99% similarity to the V5-V6 region of the 16S rRNA gene in the SILVA 138 database to match the 784F/1061R primer pair used [11]. Mitochondria and chloroplast reads were filtered out. Analyses were performed using Rstudio version 2022.02.2 and the phyloseq package [12]. Metadata file, taxonomy table, phylogenetic tree and ASV table were imported into R to create a phyloseq object. Contaminant ASVs, arising from kit reagents and sample manipulation, were identified using the package decontam [13]. The function ‘isNotContaminant’ was used as it is more stringent and more adequate for low-biomass samples. One replicate for each OI2 and OI3 were removed because of heavy contamination. It is worth noting that bacteria belonging to the *Brachybacterium* genus accounted for more than 90% of the contamination (Table S1), which were contaminants of the Qiagen PCR kit previously observed [6]. For the re-analysis of previously acquired data, raw reads were downloaded from SRA (SRP150755 for X7, and SRP187380 for OI2 and OI3). Data was re-analyzed as described above. Plots were generated using GraphdPad Prism 9.

**Genome amplification of LCM samples and shotgun sequencing**

To reach sufficient quantities for shotgun sequencing, DNA samples obtained through LCM were amplified as previously described [6], using a SeqPlex DNA Amplification Kit (Sigma-Aldrich, USA), which is specifically designed for low-quantity, fragmented DNA. The same DNA samples that were used for 16S rRNA gene metabarcoding were used and, for each sample, 1 µL (OI2 and OI3) or 3 µL (X7) of each of the three replicates were pooled before amplification. Only the X7 sample yielded sufficient DNA for sequencing. Samples were then sent to the Australian Genomic Research Facility (Melbourne, Australia) for sequencing. Library preparation was performed with an IDT xGen cfDNA & FFPE DNA Library Prep kit (Integrated DNA Technologies, USA) and samples were sequenced on a NovaSeq 6000 S4 2×150 bp Flowcell Illumina platform.

**Metagenomic data analysis**

Quality control was performed using FastQC v0.11.9 [14]. Adapters, low-quality sequences (phred score < 30), as well as the first and last 10 bp were trimmed using Trim Galore v0.6.2 [15]. High-quality reads were mapped against a *P. acuta* draft genome [16] using Bowtie2 v2.4.2 with default parameters [17] to remove host-related reads from the data. Mapped reads were then removed using Samtools v1.11 [18]. Only paired-end host-removed reads were used for metagenome assembly. Metagenome assembly was carried out using MEGAHIT v1.2.9 [19] with a minimum contig length of 1000 bp and the following k-mers: 21, 33, 55, 77, 99. Contigs from kmer 99 were used for all downstream processing. Contig taxonomy was assessed using CAT/BAT v5.2.3 [20], and contigs assigned to Eukarya, Archaea, or Virus were removed. Assembled contigs were binned using the binning (with metabat2, concoct and maxbin2 tools) and bin_refinement (with >70% completeness and <10% contamination as cut-off parameters) modules of MetaWRAP v1.3.2 [22]. Bin quality and taxonomy were assessed using CheckM v1.2.2 [23] and GTDB-Tk v2.3.0 [24], respectively. These bins were reassembled to further improve the contiguity and bin completeness and contamination stats using the reassemble_bins module implemented in MetaWRAP v1.3.2. The final taxonomy of individual contigs on a per bin level was assessed using CAT/BAT v5.2.3 [20], and any contig belonging to a different phylum than the taxonomy assigned by GTDB-Tk was manually removed. Bin coverage was obtained using CoverM v0.6.1 (https://github.com/wwood/CoverM) using the “genome” option. The full sequence of the 16S rRNA gene (1569 bp) was obtained using Barrnap v0.9 [25].

**Pure culturing of bacteria from *Pocillopora acuta***

Using clean bone cutters, fragments between 3 and 7 cm in length were sampled from the P3 colony, rinsed in FSW, and placed in sterile zip-lock bags containing FSW. Using a water flosser (Waterpik® Water Flosser) fitted with a clean nozzle, coral tissue was blasted off the skeleton. The resulting slurry was collected in a sterile zip-lock bag and transferred to 50 mL Falcon tubes. The tubes were centrifuged at 3750 × *g* for 10 min (Allegra X-12R, Beckman Coulter, USA) to obtain coral tissue pellets. The pellets were resuspended in 1 mL FSW and transferred to a single 1.5 mL tube per colony and centrifuged at 5000 × *g* for 10 min to remove any residual mucus from the sample. The tissue pellets were resuspended in 1 ml of FSW using a tissue lyser (Tissue-Lyser II, Qiagen, Australia) and the resuspension was homogenized using sterile glass homogenizers for 30 s. The resulting tissue homogenates were each serially diluted from 10^-1^ to 10^-7^ and plated in triplicates onto Marine Agar 2216 (MA, BD Difco). Following one week of incubation at 23°C, individual bacterial colonies were selected and streaked onto fresh MA plates. Purified bacterial isolates were obtained after a further 2 rounds of sub-culturing onto fresh MA plates following a weekly incubation period at 25°C and 26°C, respectively.

**16S rRNA gene sequencing of cultured bacteria**

Individual, pure, freshly grown bacterial colonies were suspended in 20 μL sterile Milli-Q® water, incubated for 10 min at 95°C then used as templates in colony PCRs. PCR amplification of the bacterial 16S rRNA gene was with primers 27F and 1492R [26]. The PCR was performed with 20 µl Mango Mix™ (Bioline, UK), 0.25 µM of each primer and 2 µl of DNA template in a final volume of 40 µl with nuclease free water (Ambion, Thermo Fisher Scientific Inc., TX, USA). The thermal cycling protocol was as follows: 94°C for 5 min; 30 cycles of 94°C for 1 min, 50°C for 45 s and 72°C for 90 s; and a final extension of 10 min at 72°C. Amplicons were purified and sequenced on an ABI sequencing instrument by Macrogen (Seoul, South Korea). Trimmed high-quality read data from each isolate were used for presumptive identification by querying the 16S rRNA gene sequences via Blastn and compared to other 16S rRNA gene sequences obtained in this study.

**DNA extraction and whole-genome sequencing**

The P3-11-1 isolate was selected for whole-genome sequencing. A single colony was picked with an inoculation loop and DNA extraction was performed on the QIAsymphony using the DSP Virus/Pathogen Mini Kit (Qiagen). Library preparation performed using Nextera XT (Illumina Inc.) according to manufacturer’s instructions. Whole-genome sequencing was performed on NextSeq 500/550 with a 150bp PE kit.

### **Genome assembly, taxonomy, and annotation**

Raw reads were trimmed and quality-filtered using Trimmomatic v0.36 [27] (HEADCROP:10 LEADING:5 TRAILING:5 SLIDINGWINDOW:4:28 MINLEN:30). The quality of raw reads before and after trimming was checked with FASTQC v0.11.9 [14]. Trimmed and quality-filtered reads were de novo assembled into contigs using SPAdes v3.15.5 [28] with 21, 33, 55, 77 and 99 k-mers, and the option “--careful” was applied to minimise the number of mismatches and short indels. All contigs with a length of <1000 bp were removed using BBMap v38.96 [29]. Subsequently, the levels of completeness and contamination of assembled genomes were assessed using CheckM v1.2.2 using the “lineage_wf” workflow [23]. Coverage was obtained using CoverM v0.6.1 (<https://github.com/wwood/CoverM>) using the “genome” option.

Taxonomic assignment of all assembled genomes was carried out using GTDB-Tk v2.3.0 [24] using the “classify_wf” workflow. GTDB-Tk assigned the taxonomy of the genomes based on 120 bacterial marker genes. Based on the GTDB-Tk alignment of the two genomes from this study and 28 additional genomes (Table S5), a phylogenetic tree was built in IQ-Tree v2.2.2.3 [30] using the best model Q.insect+F+R4, selected by ModelFinder wrapped in IQ-tree [31], and 1000 ultrafast bootstrap replicates [32]. The tree was visualized in iTOL v6 [33]. *Zookishella* was chosen as an outgroup. Average Nucleotide Identities (ANI) and Average Amino acid Identities (AAI) were calculated using a genome-based matrix calculator [34] and plotted in R using the pheatmap package [35]. Gene prediction was performed in Bakta v1.7.0 [36], KEGG-mapper Reconstruct [37], and InterProScan v5.55 with Pfam domain annotations [38]. Pfam annotations were used to look for eukaryotic-like proteins (ankyrin-repeat domains, WD40 domains, tetratricopeptide repeat) and the *dsyB* gene (PF00891). Secondary metabolites were predicted using antiSMASH v7.0.0 [39].

The 16S rRNA gene phylogenetic tree of the *Endozoicomonadaceae* family was constructed using 38 published, full-length 16S rRNA gene sequences from the *Endozoicomonadaceae* family. The full-length 16S rRNA gene (1572 bp) obtained from the P3-11-1 assembly was used for the phylogenetic tree construction. A MAFFT alignment was created using Geneious Prime v2019.1.3. The full alignment was stripped of columns containing 99% or more gaps. This alignment was used to generate a maximum likelihood phylogenetic tree with 1000 ultrafast bootstraps using IQ-Tree v2.2.2.3 [30] with the best model TIM3+F+I+G4, selected by ModelFinder wrapped in IQ-tree [31]. Sequences belonging to the *Zookishella* genus were chosen as an outgroup.

**
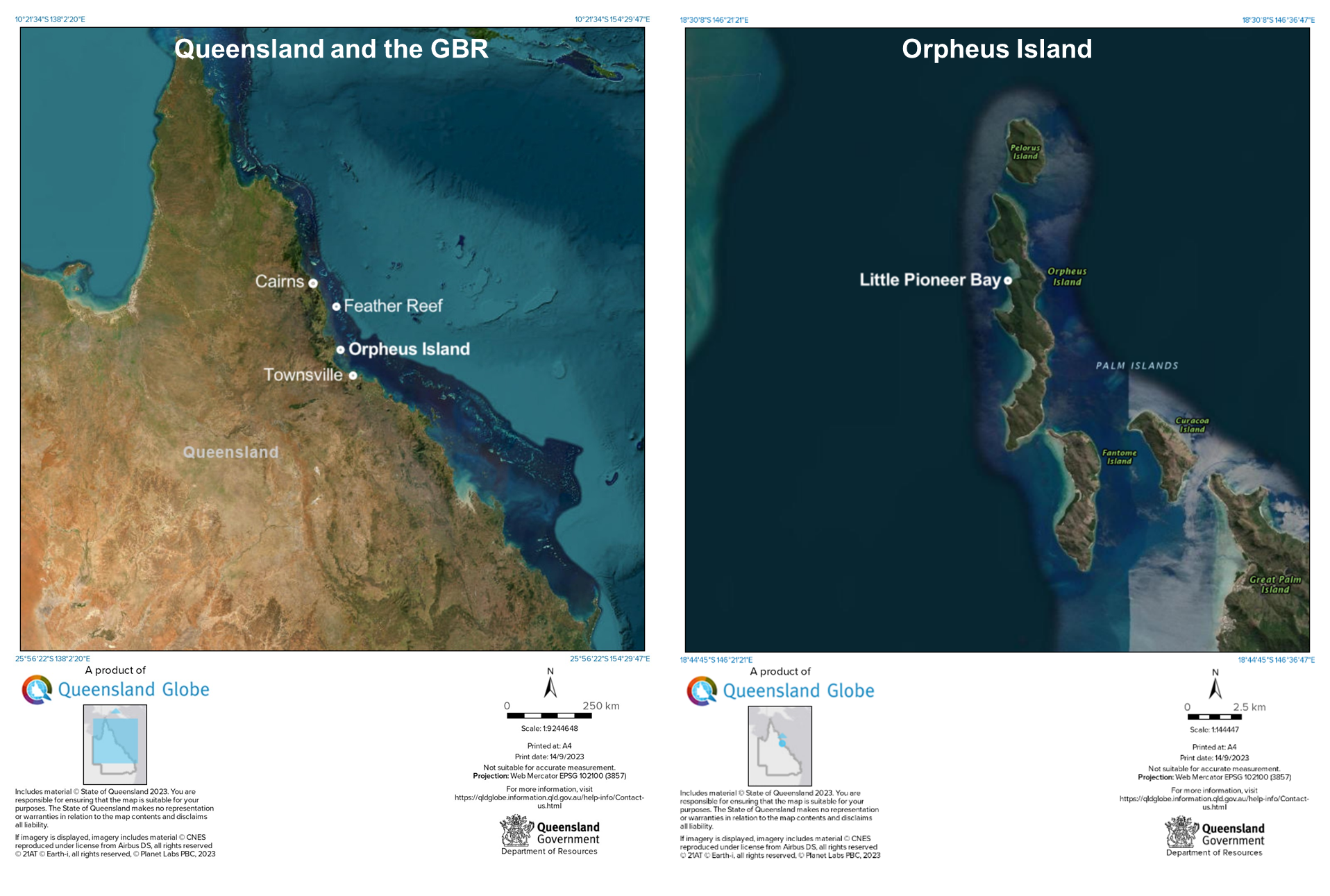
**

**Figure S1:** Map of study site at Little Pioneer Bay (Orpheus Island) in the central Great Barrier Reef. Feather Reef is also highlighted as it was the site of a previous study on CAMAs in *Pocillopora acuta* [6]*.* **© The State of Queensland (Department of Resources) 2023**

**
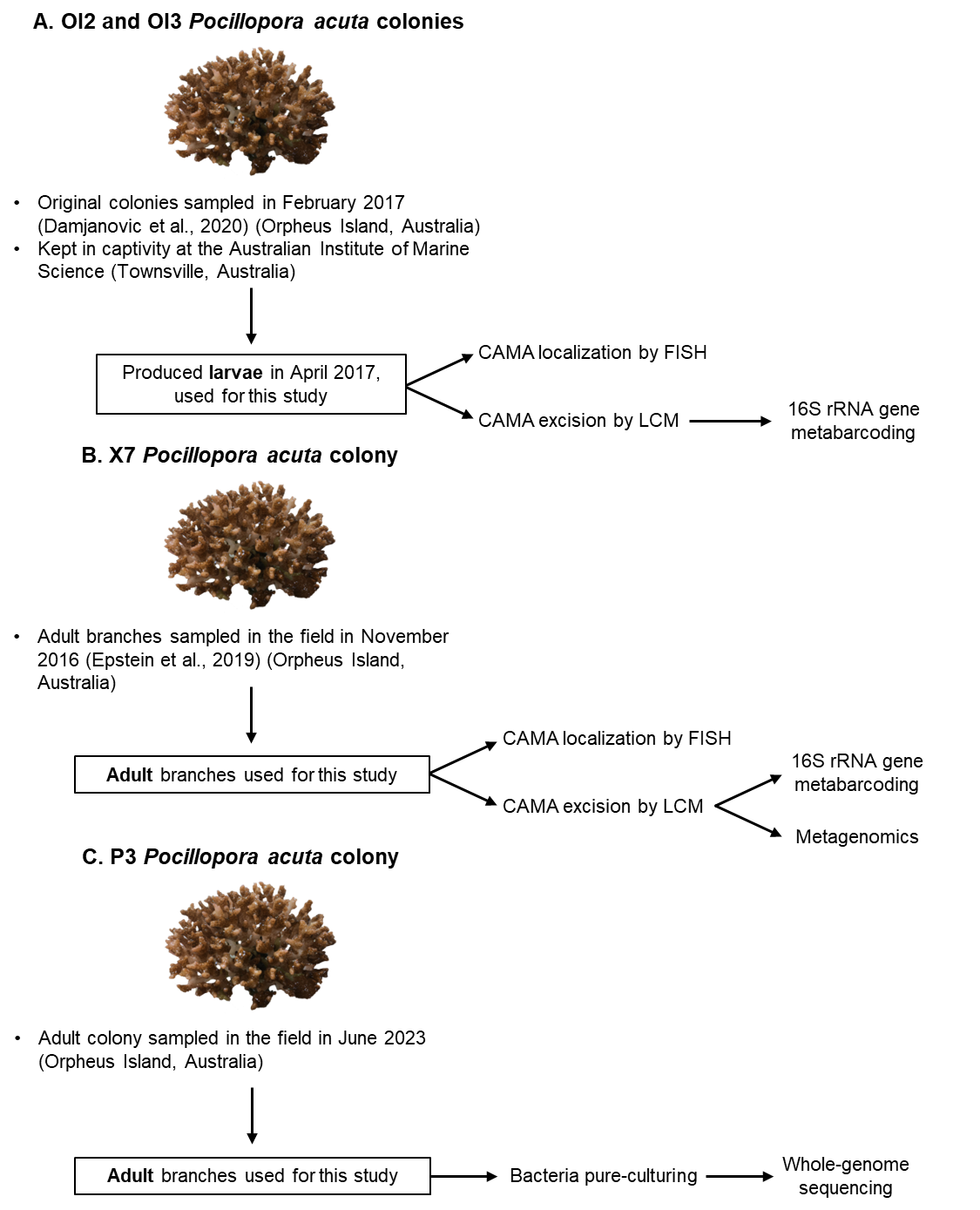
**

**Figure S2:** Sampling and experimental design used in this study. All *Pocillopora acuta* colonies were sampled from the same site in Little Pioneer Bay, Orpheus Island (Great Barrier Reef, Australia). A: Sampling of the OI2 and OI3 colonies (by Damjanovic et al. (2020) [1]). Following the establishment of the original colonies in captivity, larvae released by the adults were sampled. Larvae were used for microscopic imaging and 16S rRNA gene metabarcoding of CAMAs. In the original study, the adults and recruits were also sampled. B: Sampling of the X7 colony (by Epstein et al. (2019) [2]). Adult branches were sampled in the field and fixed. Branches were used for microscopic imaging, and 16S rRNA gene metabarcoding and metagenomics of CAMAs. C: Sampling of the P3 colony. Adult colonies were sampled in the field, and live fragments were used for the pure-culturing of bacteria and whole-genome sequencing of the isolated bacteria. FISH: fluorescence in situ hybridization; CAMA: cell-associated microbial aggregate; LCM: laser capture microdissection

**
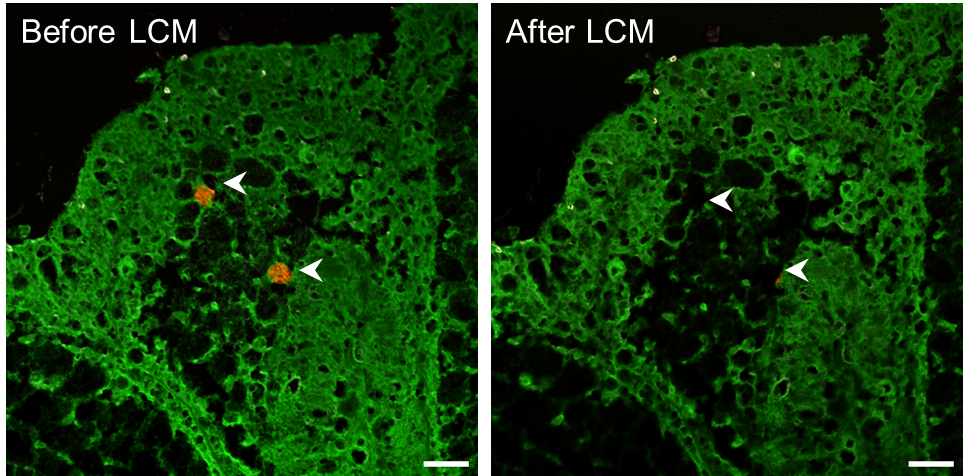
**

**Figure S3:** Laser capture microdissection (LCM) of CAMAs in *Pocillopora acuta*. Arrowheads point at CAMAs (left panel) and captured CAMAs (right panel) in the same section. Green: autofluorescence; red: EUB338-mix probe (all bacteria); white: non-EUB probe (negative control). Scale bars: 20 µm.

**
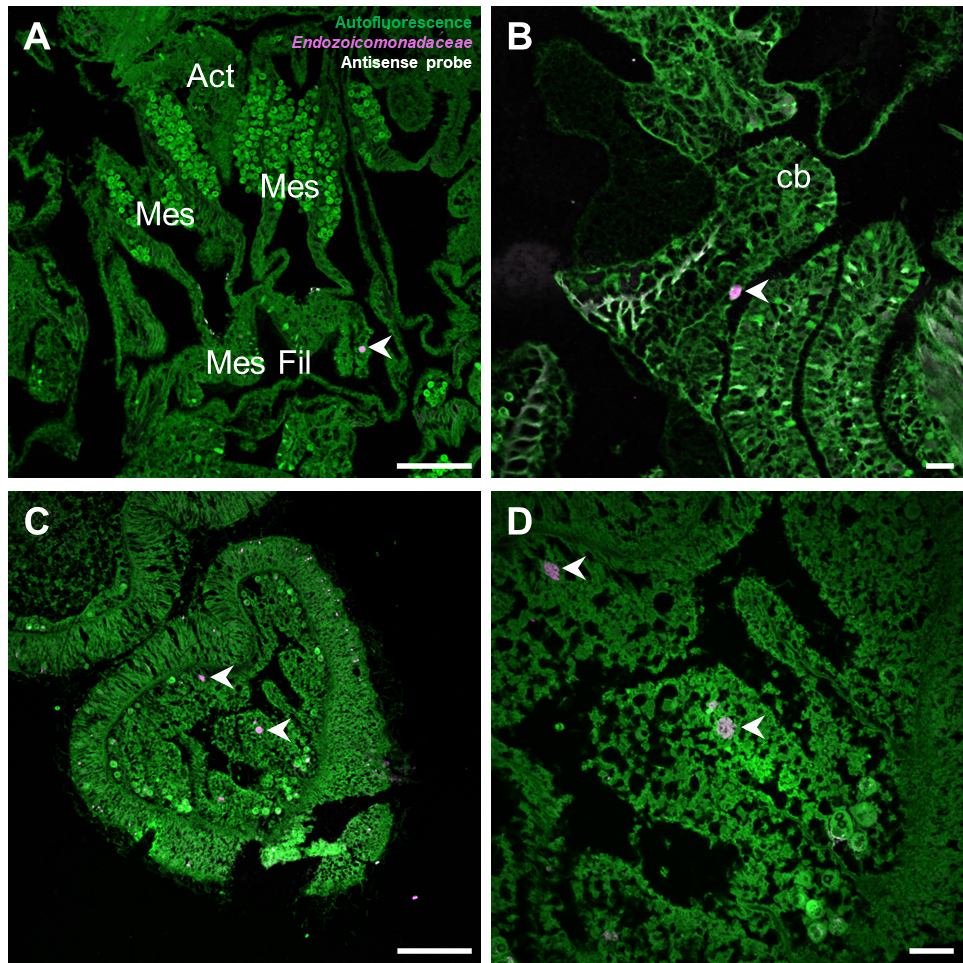
**

**Figure S4:** *Endozoicomonadaceae* in CAMAs of Orpheus Island *Pocillopora acuta*. CAMA location by FISH on sectioned adult polyps (A, B) and larvae (C, D). Arrowheads point at CAMAs. Green: autofluorescence; magenta: End663 probe (*Endozoicomonadaceae*); white: non-EUB probe (negative control). Act: actinopharynx; Mes Fil: mesenterial filaments; Mes: mesenteries; cb: cnidoglandular band. Scale bars: 100 µm for A, C; 20 µm for B, D.


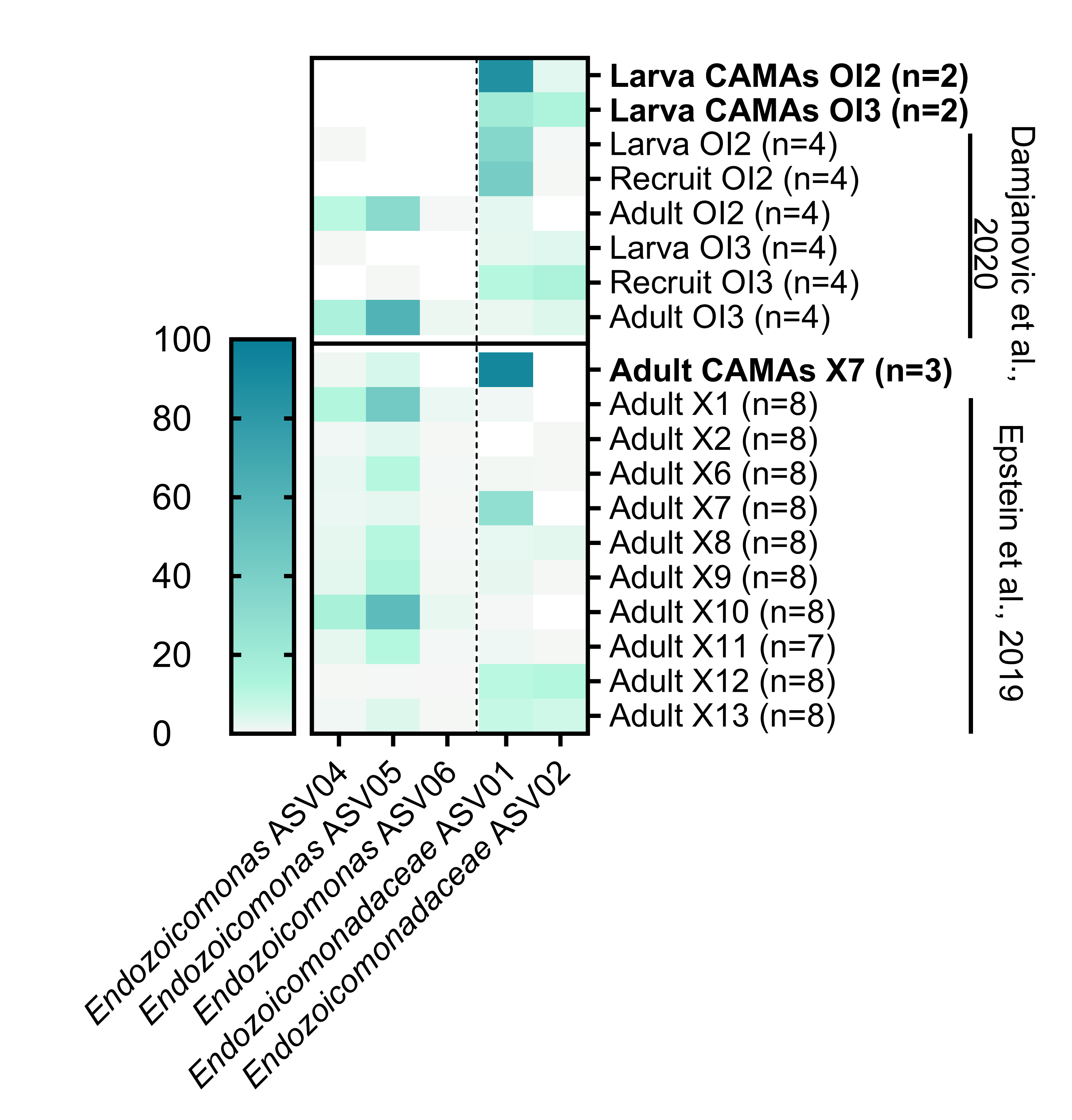


**Figure S5:** Relative abundance of the five most abundant *Endozoicomonadaceae* ASVs in CAMAs, whole larvae, recruits, and adults of Orpheus Island *Pocillopora acuta*. Rows in bold (CAMAs) are newly obtained data. Other rows represent reanalyzed data from Damjanovic et al., 2020 [4] and Epstein et al., 2019 [5]. *Endozoicomonadaceae* ASVs 01 and 02 were assigned as *Kistimonas* in our reanalysis, although they were initially assigned to *Endozoicomonas* in the original studies. Raw data are available in Table S2 (for CAMAs) and Table S3 (for the reanalysis of previous data).

**
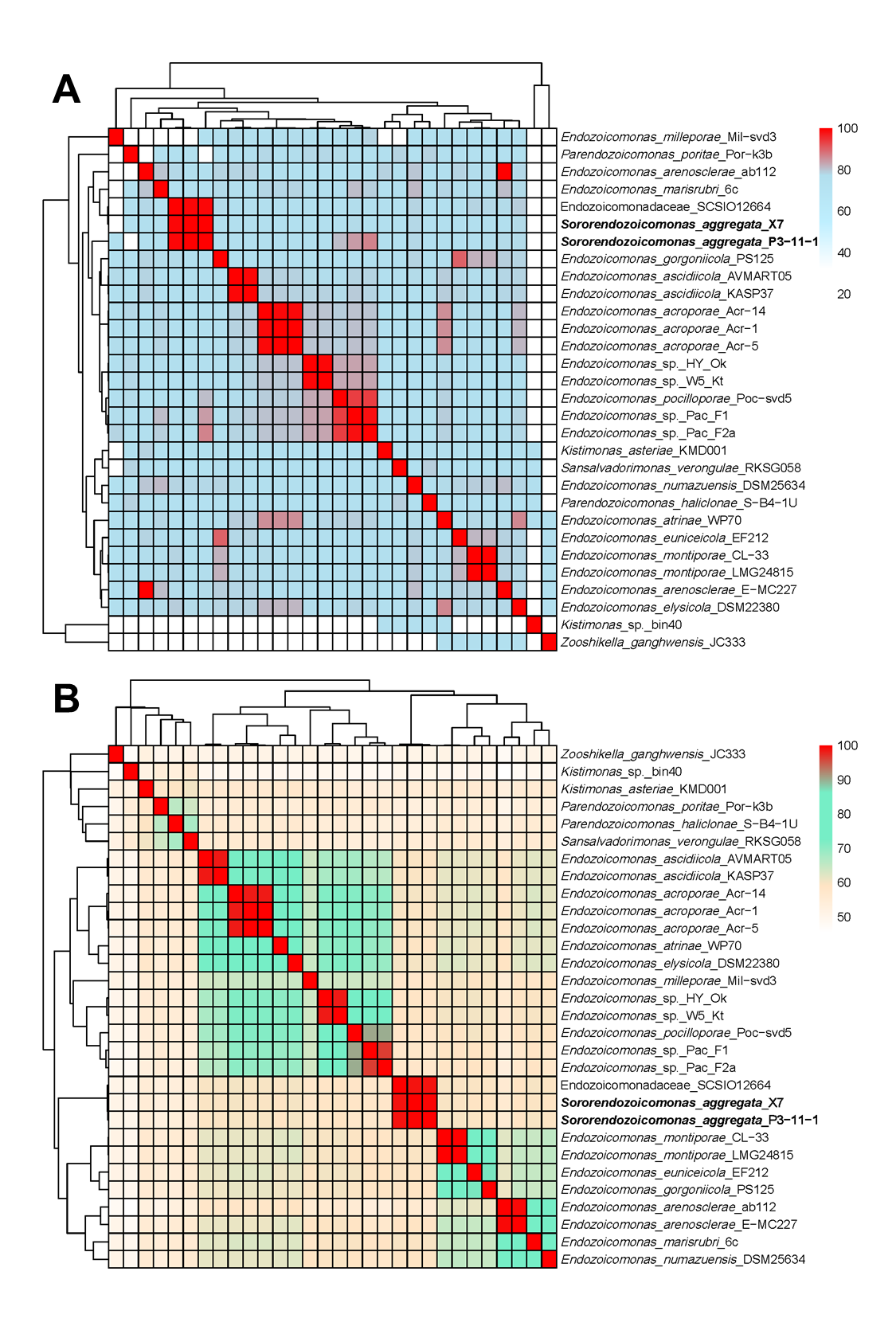
**

**Figure S6:** Average nucleotide identity (ANI) (A) and average amino acid identity (AAI) (B) of Pac_X7 and Pac_P3-11-1 with other *Endozoicomonadaceae* genomes. Additional data on the reference genomes are available in Table S5.


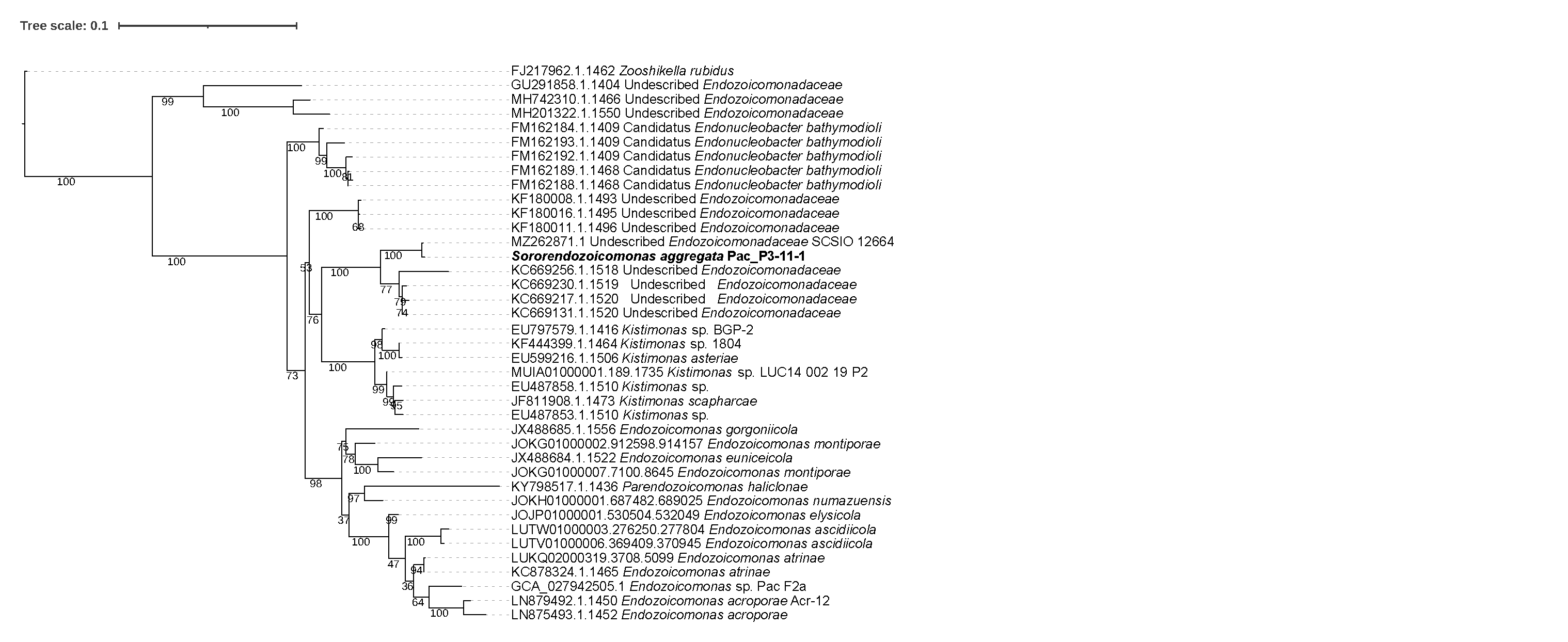


**Figure S7:** Maximum likelihood phylogenetic tree of 16S rRNA gene sequences showing the placement of Pac_P3-11-1 (in bold) in the *Endozoicomonadaceae* family based on 38 bacterial 16S rRNA sequences, in addition to the full 16S rRNA sequence obtained in this study. Bootstraps values based on 1000 replications are provided.


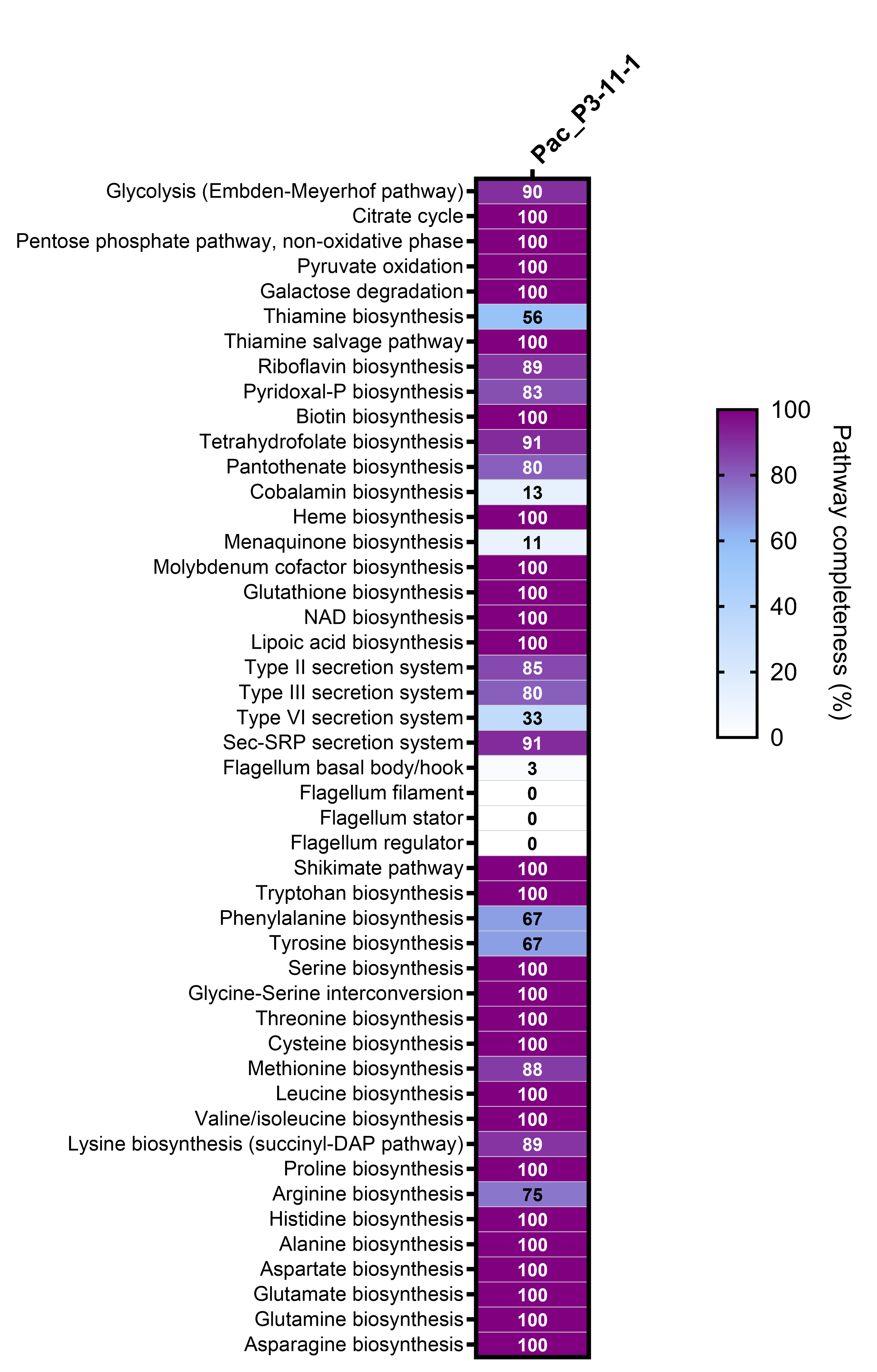


**Figure S8:** Estimated completeness of KEGG pathways of interest in the Pac_P3-11-1 genome recovered in this study.

**Table S1:** Sequencing statistics for the two 16S rRNA gene metabarcoding experiments analyzed in this study and shown in Figure 1F. The first column (“Larva CAMAs”) refers to Table S2A, the second column (“Adult CAMAs”) refers to Table S2B.

| **Experiment** | **Larva CAMAs (OI2 and OI3 colonies)** | **Adult CAMAs (X7 colony)** |
| --- | --- | --- |
| **Total Samples (negative controls)** | 18 (12) | 11 (8) |
| **Raw reads** | 1323009 | 2426491 |
| **Reads after merging, denoising and chimera filtering** | 941134 | 1755498 |
| **Samples kept for analysis** | 4 | 3 |
| **ASVs after decontamination** | 18 | 12 |
| **Read per sample** | 13647 | 74591 |
| **Contamination (%)** | 82.9 | 29.4 |
| **Proportion of *Brachybacterium* sp. among contaminants (%)** | 93.2 | 89.8 |

**Table S2:** Relative abundance of bacterial ASVs in CAMAs isolated by LCM from larvae of the OI2 and OI3 colonies (A) and from adults of the X7 colony (B). Each column is a biological replicate. These data are summarized in Figure 1F.

(attached)

**Table S3:** Relative abundance of *Endozoicomonadaceae* ASVs in larvae, recruits, and adults of the OI2 and OI3 colonies (A), and adults of 10 colonies, including X7, from Orpheus Island based on a reanalysis of data reported in Damjanovic et al. (2020) [4] and Epstein et al. (2019) [5], respectively. Each column is the average of four (A) or seven to eight (B) biological replicates. These data are summarized in Figure S5.

(attached)

**Table S4:** Summary of the two assemblies recovered from bacterial pure cultures (Pac_P3-11-1) and from X7 CAMAs (Pac_X7)

| **Genome** | **Pac_P3-11-1** | **Pac_X7 (MAG)** |
| --- | --- | --- |
| **Size (bp)** | 4041637 | 3926126 |
| **Coverage (X)** | 61 | 37221 |
| **Completeness (%)** | 97.13 | 95.91 |
| **Contamination (%)** | 0.88 | 1.01 |
| **G+C content (%)** | 46.7 | 46.7 |
| **N50** | 37409 | 9295 |
| **L50** | 35 | 132 |
| **Number of contigs** | 201 | 604 |
| **Number of coding sequences (CDSs)** | 3545 | 3398 |
| **Number of ribosomal RNAs** | 4 | 3 |
| **Number of transfer RNAs** | 56 | 48 |

**Table S5:** List of *Endozoicomonadaceae* genomes used for phylogenetic analyses.

(attached)

**Table S6:** Detailed Bakta annotations for the Pac_P3-11-1 genome recovered in this study.

(attached)

**Table S7:** Number of eukaryotic-like protein sequences found in the Pac_P3-11-1 genome. Sequences were detected based on an InterProScan classification with Pfam domain annotations.

| **Genome** | **Pac_P3-11-1** |
| --- | --- |
| Ankyrin-repeat proteins | 12 |
| WD40 domain proteins | 14 |
| Tetratricopeptide repeat proteins | 9 |

**Table S8:** List of predicted secondary metabolites in the Pac_P3-11-1 genome recovered in this study.

| **Contig name** | **Type** | **Most similar known cluster** | **Biosynthetic gene cluster** | **Similarity (%)** | **Prokka annotation** |
| --- | --- | --- | --- | --- | --- |
| contig_6 | RiPP-like | - | - | - | AP-endonuc-2 domain-containing protein |
| contig_24 | betalactone | fengycin | BGC0001095 | 13% | Acetyl-CoA synthetase |
| contig_37 | NI-siderophore | fulvivirgamide A2/fulvivirgamide B2/fulvivirgamide B3/fulvivirgamide B4 | BGC0002620 | 66% | Siderophore synthetase component |

**Table S9:** List of oligonucleotides probes used for Fluorescence *in situ* Hybridization.

| **Target group** | **Probe (fluorophore)** | **Sequence (5'-3')** | **% Formamide** | **NaCl concentration in washing buffer (M)** | **Ref** |
| --- | --- | --- | --- | --- | --- |
| All bacteria | EUB338-mix (atto550) | GCWGCCWCCCGTAGGWGT | 25 | 0.149 | [40] |
| Negative control | nonEUB (atto647) | ACATCCTACGGGAGG | 25-35 | 0.07 - 0.149 | [41] |
| *Endozoicomondaceae* | End663 (atto550) | GGAAATTCCACACTCCTC | 35 | 0.07 | [3] |

**Supplementary references**

1. Hedlund BP, Chuvochina M, Hugenholtz P, Konstantinidis KT, Murray AE, Palmer M, et al. SeqCode: a nomenclatural code for prokaryotes described from sequence data. *Nat Microbiol* 2022; **7**: 1702–1708.

2. Li J, Zou Y, Yang J, Li Q, Bourne DG, Sweet M, et al. Cultured Bacteria Provide Insight into the Functional Potential of the Coral-Associated Microbiome. *mSystems* 2022; **7**: e00327-22.

3. Bayer T, Neave MJ, Alsheikh-Hussain A, Aranda M, Yum LK, Mincer T, et al. The microbiome of the red sea coral stylophora pistillata is dominated by tissue-associated endozoicomonas bacteria. *Applied and Environmental Microbiology* 2013; **79**: 4759–4762.

4. Damjanovic K, Menéndez P, Blackall LL, van Oppen MJH. Mixed-mode bacterial transmission in the common brooding coral Pocillopora acuta. *Environmental Microbiology* 2020; **22**: 397–412.

5. Epstein HE, Torda G, van Oppen MJH. Relative stability of the Pocillopora acuta microbiome throughout a thermal stress event. *Coral Reefs* 2019; **38**: 373.

6. Maire J, Tandon K, Collingro A, van de Meene A, Damjanovic K, Gotze CR, et al. Colocalization and potential interactions of Endozoicomonas and chlamydiae in microbial aggregates of the coral Pocillopora acuta. *Science Advances* 2023; **9**: eadg0773.

7. Maire J, Blackall LL, van Oppen MJH. Microbiome characterization of defensive tissues in the model anemone Exaiptasia diaphana. *BMC Microbiology* 2021; **21**: 152.

8. Bolyen E, Rideout JR, Dillon MR, Bokulich NA, Abnet CC, Al-Ghalith GA, et al. Reproducible, interactive, scalable and extensible microbiome data science using QIIME 2. *Nature Biotechnology* 2019; **37**: 852–857.

9. Martin M. Cutadapt removes adapter sequences from high-throughput sequencing reads. *EMBnet.journal* 2011; **17**: 10.

10. Callahan BJ, McMurdie PJ, Rosen MJ, Han AW, Johnson AJA, Holmes SP. DADA2: High-resolution sample inference from Illumina amplicon data. *Nature Methods* 2016; **13**: 581–583.

11. Bokulich NA, Kaehler BD, Rideout JR, Dillon M, Bolyen E, Knight R, et al. Optimizing taxonomic classification of marker-gene amplicon sequences with QIIME 2’s q2-feature-classifier plugin. *Microbiome* 2018; **6**: 90.

12. McMurdie PJ, Holmes S. phyloseq: An R package for reproducible interactive analysis and graphics of microbiome census data. *PLoS ONE* 2013; **8**: e61217.

13. Davis NM, Proctor DiM, Holmes SP, Relman DA, Callahan BJ. Simple statistical identification and removal of contaminant sequences in marker-gene and metagenomics data. *Microbiome* 2018; **6**: 226.

14. Andrews S. FASTQC. A quality control tool for high throughput sequence data. https://www.bioinformatics.babraham.ac.uk/projects/fastqc/. Accessed 17 Oct 2022.

15. Krueger F, James F, Ewels P, Afyounian E, Weinstein M, Schuster-Boeckler B, et al. TrimGalore: v0.6.10 - add default decompression path. 2023. Zenodo.

16. Vidal-Dupiol J, Chaparro C, Pratlong M, Pontarotti P, Grunau C, Mitta G. Sequencing, de novo assembly and annotation of the genome of the scleractinian coral, Pocillopora acuta. *bioRxiv* 2020; 698688.

17. Langmead B, Salzberg SL. Fast gapped-read alignment with Bowtie 2. *Nature methods* 2012; **9**: 357–9.

18. Li H, Handsaker B, Wysoker A, Fennell T, Ruan J, Homer N, et al. The Sequence Alignment/Map format and SAMtools. *Bioinformatics* 2009; **25**: 2078–2079.

19. Li D, Liu CM, Luo R, Sadakane K, Lam TW. MEGAHIT: an ultra-fast single-node solution for large and complex metagenomics assembly via succinct de Bruijn graph. *Bioinformatics (Oxford, England)* 2015; **31**: 1674–1676.

20. Von Meijenfeldt FAB, Arkhipova K, Cambuy DD, Coutinho FH, Dutilh BE. Robust taxonomic classification of uncharted microbial sequences and bins with CAT and BAT. *Genome Biology* 2019; **20**: 1–14.

21. Gurevich A, Saveliev V, Vyahhi N, Tesler G. QUAST: Quality assessment tool for genome assemblies. *Bioinformatics* 2013; **29**: 1072–1075.

22. Uritskiy GV, Diruggiero J, Taylor J. MetaWRAP - A flexible pipeline for genome-resolved metagenomic data analysis. *Microbiome* 2018; **6**: 158.

23. Parks DH, Imelfort M, Skennerton CT, Hugenholtz P, Tyson GW. CheckM: assessing the quality of microbial genomes recovered from isolates, single cells, and metagenomes. *Genome Research* 2015; **25**: 1043–1055.

24. Chaumeil PA, Mussig AJ, Hugenholtz P, Parks DH. GTDB-Tk: a toolkit to classify genomes with the Genome Taxonomy Database. *Bioinformatics* 2020; **36**: 1925–1927.

25. Seemann T. barrnap 0.9: rapid ribosomal RNA prediction. https://github.com/tseemann/barrnap. .

26. Lane D. 16S/23S rRNA sequencing. In: Stackebrandt E, Goodfellow M (eds). *Nucleic Acid techniques in bacterial systematics*. 1991. John Wiley & Sons, New York, pp 115–47.

27. Bolger AM, Lohse M, Usadel B. Trimmomatic: a flexible trimmer for Illumina sequence data. *Bioinformatics (Oxford, England)* 2014; **30**: 2114–2120.

28. Bankevich A, Nurk S, Antipov D, Gurevich AA, Dvorkin M, Kulikov AS, et al. SPAdes: a new genome assembly algorithm and its applications to single-cell sequencing. *J Comput Biol* 2012; **19**: 455–477.

29. Bushnell B, Rood J, Singer E. BBMerge – Accurate paired shotgun read merging via overlap. *PLOS ONE* 2017; **12**: e0185056.

30. Minh BQ, Schmidt HA, Chernomor O, Schrempf D, Woodhams MD, Von Haeseler A, et al. IQ-TREE 2: New Models and Efficient Methods for Phylogenetic Inference in the Genomic Era. *Molecular Biology and Evolution* 2020; **37**: 1530–1534.

31. Kalyaanamoorthy S, Minh BQ, Wong TKF, von Haeseler A, Jermiin LS. ModelFinder: fast model selection for accurate phylogenetic estimates. *Nat Methods* 2017; **14**: 587–589.

32. Hoang DT, Chernomor O, von Haeseler A, Minh BQ, Vinh LS. UFBoot2: Improving the Ultrafast Bootstrap Approximation. *Molecular Biology and Evolution* 2018; **35**: 518–522.

33. Letunic I, Bork P. Interactive Tree Of Life (iTOL) v5: an online tool for phylogenetic tree display and annotation. *Nucleic Acids Research* 2021; **49**: W293–W296.

34. Rodriguez-R LM, Konstantinidis KT. The enveomics collection: a toolbox for specialized analyses of microbial genomes and metagenomes. 2016.

35. Kolde R. Pheatmap: pretty heatmaps. 2017.

36. Schwengers O, Jelonek L, Dieckmann MA, Beyvers S, Blom J, Goesmann A. Bakta: rapid and standardized annotation of bacterial genomes via alignment-free sequence identification. *Microb Genom* 2021; **7**: 000685.

37. Kanehisa M, Sato Y. KEGG Mapper for inferring cellular functions from protein sequences. *Protein Sci* 2020; **29**: 28–35.

38. Jones P, Binns D, Chang HY, Fraser M, Li W, McAnulla C, et al. InterProScan 5: genome-scale protein function classification. *Bioinformatics* 2014; **30**: 1236–1240.

39. Blin K, Shaw S, Augustijn HE, Reitz ZL, Biermann F, Alanjary M, et al. antiSMASH 7.0: new and improved predictions for detection, regulation, chemical structures and visualisation. *Nucleic Acids Research* 2023; **51**: W46–W50.

40. Daims H, Brühl A, Amann R, Schleifer KH, Wagner M. The domain-specific probe EUB338 is insufficient for the detection of all bacteria: Development and evaluation of a more comprehensive probe set. *Systematic and Applied Microbiology* 1999; **22**: 434–444.

41. Wallner G, Amann R, Beisker W. Optimizing fluorescent in situ hybridization with rRNA-targeted oligonucleotide probes for flow cytometric identification of microorganisms. *Cytometry* 1993; **14**: 136–143.
